# Parenting stress: a cross-sectional analysis of associations with childhood obesity, physical activity, and TV viewing

**DOI:** 10.1186/1471-2431-14-244

**Published:** 2014-10-01

**Authors:** Kathryn Walton, Janis Randall Simpson, Gerarda Darlington, Jess Haines

**Affiliations:** Department of Family Relations and Applied Nutrition, University of Guelph, 50 Stone Road East, N1G 2 W1 Guelph, Ontario Canada; Department of Mathematics and Statistics, University of Guelph, 50 Stone Road East, N1G 2W1 Guelph, Ontario Canada

**Keywords:** Childhood, Obesity, Preschoolers, TV viewing, Physical activity, Parenting, Stress, Home environment

## Abstract

**Background:**

Parents influence their children’s obesity risk through feeding behaviours and modeling of weight-related behaviours. Little is known about how the general home environment, including parental stress, may influence children’s weight. The objective of this study was to explore the association between parenting stress and child body mass index (BMI) as well as obesity risk factors, physical activity and television (TV) viewing.

**Methods:**

We used cross-sectional data from 110 parent–child dyads participating in a community-based parenting intervention. Child heights and weights were measured by trained research assistants. Parents (93% mothers) reported level of parenting stress via the Parenting Stress Index- Short Form (PSI-3-SF) as well as children’s activity behaviours and TV viewing. This was an ethnically diverse (55% Hispanic/Latino, 22% Black), low-income (64% earning < $45,000/year) sample.

**Results:**

Level of parenting stress was not associated with children’s risk of being overweight/obese. Children with highly stressed parents were less likely to meet physical activity guidelines on weekdays than children with normally stressed parents (OR = 0.33, 95% CI, 0.12-0.95). Parents experiencing high stress were less likely to set limits on the amount of TV their children watched (OR = 0.32, 95% CI, 0.11, 0.93).

**Conclusion:**

Results suggest stress specific to parenting may not be associated with increased obesity risk among children. However, future interventions may need to address stress as a possible underlying factor associated with unhealthful behaviours among preschoolers.

## Background

Approximately 21% of American preschoolers aged 2–5 years are overweight or obese, bringing childhood obesity to the forefront of public health concern [1]. Among preschoolers, intrapersonal factors such as diet and physical activity habits, along with certain parental characteristics and behaviours (e.g. parental obesity, parent feeding behaviours, parenting style), are known to increase obesity risk [2, 3]. Less is known about how the general family environment, such as level of stress in the home, influences children’s obesity risk.

Stress is associated with the release of the steroid cortisol [4, 5]. Emerging evidence shows that enhanced levels of stress, associated with chronically high levels of cortisol, can lead to neuro-endocrine responses that alter metabolism, appetite and activity levels and, consequently, obesity risk in both adults and children [4–6]. Stress specific to parenting has been defined by a complex construct involving behavioural, cognitive and affective components relating to a person’s appraisal of his or her role as a parent [7–9]. Research suggests that high parenting stress may lead to increased obesity risk among children in two ways, 1) triggering the child’s own physiological response to stress and 2) parent stress may lead to compromised parenting which promotes unhealthful behaviours. Stress may culminate in a lack of time spent with the child, which may cause higher physiological stress levels among children, resulting in higher obesity risk through alterations to neuro-endocrine responses [10]. Parenting stress may also influence children’s weight-related behaviours; parents experiencing higher levels of stress may feel they lack the time or energy to be physically active with their children or to model such active behaviours [11]. These stressed parents may also cope by keeping their children occupied watching television [3].

To date, few studies have examined the association between stress in the home environment and child weight; with the majority of studies focused on general family stressors versus stress specific to parenting [10, 12–14]. In their cross-sectional study of 3–17 year olds, Parks et al. [14] found that the number of general family stressors present (financial strain, mental and physical health and family structure) was associated with increased odds of child overweight and obesity in the total sample (OR = 1.26, 95% CI, 1.18-1.35). However, this association did not hold for the parents of preschoolers when stratified by age (OR = 1.05, 95% CI, 0.84-1.31) [14]. Similarly, Garasky et al. [12] found an association between financial strain and obesity risk among older children (aged 12–17 years), but not among younger children (aged 5–11 years) [12]. A single study has examined the influence of parenting stress specifically on children’s obesity risk; Koch et al. [13] examined the association between 4 domains of stress (serious life events, parenting stress, lack of social support and parent worries) and child weight among a predominantly white sample of families and found that the children of parents who reported at least 2 of their 4 domains of stress had an increased odds of obesity both cross-sectionally at age 2 (OR = 2.55, 95% CI, 1.46, 4.46) and longitudinally at age 5 (OR = 3.32, 95% CI, 1.39, 7.90). When exploring the relationship between individual stressors and child obesity risk, however, Koch et al. [13] only found a significant relationship between the serious life events domain and childhood obesity; the parenting stress domain was not significantly associated with child weight. Compared to many general family stressors, (ex. financial strain or health status) which are not easily changed, stress specific to parenting is mutable; studies have demonstrated an increase in positive parenting behaviours and improved child outcomes in response to reductions in parenting stress [15]. Thus, an understanding of the association between stress specific to parenting and child weight status is needed to inform future obesity prevention efforts and interventions.

While Parks et al. [14] reported that young children of parents who self-reported high general stress levels ate more fast food than the children of parents who did not report high levels of stress, little is known about how stress may affect other weight-related behaviours, such as physical activity and TV viewing. Lampard and colleagues [16] examined family level factors associated with limiting screen time among a low-income sample of parents of young children and found that parents with lower parenting stress were more likely to limit their child’s screen time, suggesting that parenting stress may impact a parent’s ability to provide his/her child with a home environment that encourages healthy behaviours.

The current study builds on this small body of literature by examining how parenting stress is associated with childhood obesity and related behaviours among an ethnically diverse sample of preschool aged children. Our primary objective was to examine the association between parenting stress and child body mass index (BMI). We hypothesized that parental stress would be associated with children’s measured BMI level. Our second objective was to assess how unhealthful behaviours known to be associated with increased obesity in children, e.g., physical activity and television viewing [17, 18], might be affected by parenting stress level. We hypothesized that parenting stress level would be directly associated with children’s TV viewing, and inversely associated with parental efforts to limit child TV time and child physical activity.

## Methods

### Study design and participants

We conducted secondary data analyses using baseline data from the Parents and Tots Together (PTT) study, a randomized controlled trial of a community-based parenting intervention. The current study provides a cross-sectional analysis of the association between parenting stress and child weight and weight-related behaviours in 110 parent–child dyads.

Participants were parents with preschool children between the ages of 2–5 years. Exclusion criteria for the PTT study included: 1) inability to respond to surveys in either English or Spanish; 2) plans to move from the Boston area during the study period; 3) parents who were younger than 18 years of age; and 4) children or parents with severe health conditions such as cardiac concerns or severe asthma that would inhibit them from participating in study activities. PTT is a primary prevention intervention; therefore, children were eligible regardless of their weight status. Written informed consent was obtained from all parent participants on behalf of themselves and their preschoolers. Ethical approval for the current study was obtained through the University of Guelph Ethics Review Board and the Harvard Pilgrim Health Care Human Participants Committee.

### Measures

#### Stress

Parenting stress was measured using the 12-item parent distress subscale of the Parenting Stress Index Short Form 3rd Edition (PSI-3-SF) [7, 15, 19, 20]. The distress subscale assesses the level of distress experienced by a parent, arising from life restrictions due to demands of child rearing [7]. We found the 12-items of the parent distress subscale to have good internal consistency (α = 0.906). Responses to each of the 12 items of the Parental Distress domain were summed and averaged to create an overall score of parenting stress. Total scores were then compared to the stress categories described in the PSI-3-SF manual to determine the level of stress parents were experiencing [7]. Based on their correspondence with the reference categories, parent stress scores were collapsed into two categories: normal stress vs. high stress. Those who scored above the 85th percentile on the Parent Distress Subscale of the PSI-SF were considered to be experiencing high parenting stress.

#### Outcome measures

Our primary outcome measure was BMI level. Trained research assistants measured children’s heights and weights at baseline. Based on the WHO growth charts, we calculated BMI z-scores to assess child weight across gender groups and the preschool age span. For the purpose of data analysis, we collapsed the BMI z-scores into two categories to characterize child weight: normal weight (BMI z-score ≤ +1) vs. overweight/obese (BMI z-score > +1) [21].

As secondary outcomes, we assessed child physical activity habits, child TV viewing and parental limits on child TV viewing. Child physical activity behaviours were measured based on parent-report of the amount of active play the child participated in on weekdays and weekend days: “On an average weekday [weekend day], how much time per day is your child involved in active play (such as running, jumping, climbing)” [22]. Answers were based on a 6-point Likert scale ranging from 0 minutes to 2 hours or more per day. Based on the National Association for Sport and Physical Education (NASPE) recommendation that preschoolers spend at least 60 minutes in moderate to vigorous active play per day, responses were collapsed into two categories: >1 hr/day vs. ≤1 hr/day [23]. We analyzed weekday and weekend day physical activity separately.

Child television viewing habits were measured by parent-report of a child’s habits on a typical weekday and weekend using the question “On an average weekday [weekend day] how much time per day does your child spend watching TV incl. DVDs or videos?” [24]. Based on the American Academy of Pediatrics (AAP) recommendations that preschool children spend no more than 2 hours per day watching TV and other media, viewing time was collapsed into two categories: >2 hours/day vs. ≤ 2 hours/day [25]. We analysed TV viewing time separately for weekdays and weekend days. The AAP also suggests that parents ‘limit’ the amount of television their preschoolers’ watch [25]. Parents’ use of limits on TV time was measured based on the following question “I limit the amount of time my child watches TV or videos” [22]. Responses were provided on a 4-point Likert scale (Response Answers: strongly agree, agree, disagree, and strongly disagree). For ease of interpretation, responses were collapsed into two categories: Agree or Disagree. We used the NASPE and AAP recommendations as both considered obesity prevention when developing their cut-offs [23, 25].

### Statistical analysis

All statistical analyses were conducted using SPSS version 20 for Windows (PASW, IBM, New York, USA). Data on participant demographics were analyzed by calculating means (SD) and frequencies. We used logistic regression models to examine associations between parenting stress, child weight status and weight-related behaviour outcomes. We considered odds ratios to be significant if the 95% confidence interval did not contain one [26]. Parental marital status and education attainment [27] were included in all models due to their known association with both increased stress and obesity risk [28, 29]. Both were categorized as binary variables: married/living with a partner vs. single/divorced/separated and graduated high school or less vs. some college/technical school or degree. As the preschool age group is a dynamic time of development, we also stratified our analyses by age using two groups, 2 and 3 year olds and 4 and 5 year olds to help us understand any differences in the health behaviours of interest by age (data not shown). When stratifying by age we did not find any substantive differences in our results and thus we will only be presenting data analysing our preschooler population as a whole.

## Results

Table 1 shows the baseline characteristics of the parents and children in our study sample. Of the 110 parents who completed the questionnaire, 101 (92.7%) were the biological mothers of the child participants. The majority of parents were either married (45.8%) or living with a partner (29%). This was an ethnically diverse (55.2% Hispanic/Latino, 21.9% Black), low-income sample with the majority of households earning less than $45,000/year (63.8%). Forty percent of parents had not graduated high school. Parents scored an average of 28.4 ± 10.69 on the PSI-SF parent distress subscale, which categorized 20% as experiencing high levels of stress. The mean age of the children was 3.15 years ± 1 year; 48.5% were classified as overweight or obese.

**Table 1:** **Baseline parent and child characteristics among participants in the parents and tots together intervention (N = 110)**

	N (%)
**Parent relation to child**	
**Mother**	101 (92.7%)
**Stepmother**	1 (0.9%)
**Foster-mother**	1 (0.9%)
**Father**	5 (4.6%)
**Grandparent**	1 (0.9%)
**Parent marital status**	
**Married**	49 (45.8%)
**Not married, living with a Partner**	31 (29.0%)
**Single**	22 (20.6%)
**Divorced**	2 (1.9%)
**Separated**	3 (2.8%)
**Race/Ethnicity**	
**Hispanic/Latino**	58 (55.2%)
**White**	14 (13.3%)
**Black**	23 (21.9%)
**Other**	10 (9.5%)
**Total household income**	
**< $20, 000**	34 (30.9%)
**$20,000-$45,000**	36 (32.8%)
**$46,000-$99,999**	6 (5.4%)
**$100,000 or more**	4 (3.6%)
**Don’t know/Refused**	30 (27.3%)
**Parent education obtained**	
**8th grade or less**	22 (21.2%)
**Some high school**	19 (18.3%)
**Graduated high school**	18 (17.3%)
**Some college or Technical school**	23 (22.1%)
**Post graduate rraining or degree**	21 (20.2%)
**Don’t know**	1 (1.0%)
**Parent stress level**	**Mean stress level (SD) = 28.38 (10.691)**
**Normal stress**	88 (80%)
**High stress**	22 (20%)
**Child age (years)**	**Mean age (SD) =3.15 (1.0)**
**2**	36 (32.7)
**3**	36 (32.7)
**4**	25 (22.8)
**5**	13 (11.8)
**Child gender**	
**Male**	56 (50.9%)
**Female**	54 (49.1%)
**Child BMI categorization**	
**Normal weight**	52 (51.5%)
**Overweight/Obese**	49 (48.5%)
**Child physical activity (Active Play)**	
***Weekday***	
**<1 hour/day**	36 (33.0%)
**≥ 1 hour/day**	73 (67.0%)
***Weekend day***	
**<1 hour/day**	28 (26.9%)
**≥ 1 hour/day**	76 (73.1%)
**Child TV viewing**	***Mean viewing Hrs. (SD) = 3.48 (1.040)***
***Weekday***	
**≥ 2 hour/day**	55 (51.9%)
**<2 hour/day**	51 (48.1%)
***Weekend Day***	***Mean viewing Hrs. (SD) = 3.45 (1.152)***
**≥ 2 hour/day**	56 (52.8%)
**<2 hour/day**	50 (47.2%)
**Parent limiting of TV viewing**	
**Disagree**	22 (20.0%)
**Agree**	88 (80.0%)

Level of parenting stress was not associated with child weight status (Table 2; OR = 1.01, 95% CI, 0.35- 2.91). Parenting stress level was inversely associated with active play on weekdays. Compared to the children of normally stressed parents, children with parents experiencing high stress levels were less likely to meet the preschooler physical activity guidelines on weekdays (OR for meeting guidelines on weekdays = 0.33, 95% CI, 0.12, 0.95). Parenting stress was not associated with children meeting physical activity guidelines on weekend days (OR for meeting guidelines on weekends = 0.59, 95% CI, 0.92-1.75). We did not find a significant association between parenting stress and children’s television viewing on weekdays (OR for watching 2 or more hours per day on weekdays = 0.95, 95% CI, 0.33-2.31) or weekends (OR for watching 2 or more hours per day on weekends =1.93, 95% CI, 0.66- 5.66). However, parenting stress was inversely associated with TV limiting by parents. Compared to parents with normal levels of parenting stress, those experiencing high levels of stress were less likely to set limits on their child’s TV viewing (OR for not limiting the TV = 0.32, 95% CI, 0.11-0.93).

**Table 2:** **OR estimates from logistic regression analyses of child obesity & obesity risk behaviours with parentingstress**

	Child overweight/Obese (BMI z-score > +1)	TV weekday (≥2 hrs day)	TV weekend (≥2 hrs/day)	Limit TV time	Active play weekday (≥1 hr/day)	Active play weekend (≥1 hr/day)
	N = 95	N = 103	N = 103	N = 103	N = 103	N = 103
Parenting stress level						
Normal						
	1.0	1.0	1.0	1.0	1.0	1.0
High						
*Unadjusted*	1.14	0.95	1.76	0.34	0.31	0.54
	(0.41, 3.18)	(0.37,2.45)	(0.63,4.90)	(0.12,0.96)*	(0.12, 0.82)*	(0.19, 1.58)
*Adjusted* ^*1*^	1.01	0.87	1.93	0.32	0.33	0.59
	(0.35- 2.91)	(0.33,2.31)	(0.66,5.66)	(0.11, 0.93)*	(0.12, 0.95)*	(0.92, 1.75)

Values presented are ORs (95% CI).

^1^adjusted for marital status and parent education attainment.

*CI does not contain 1.

## Discussion

Little is known about how the general home environment, including parenting stress, may influence children’s weight and related behaviours [2]. The objective of this study was to fill this gap, exploring the association between parenting stress, child BMI and behaviours that may place preschoolers at risk for increased weight gain. To our knowledge, this is the first study to examine the association between parenting stress and child weight with an ethnically diverse population.

In this cross-sectional study of 110 parent–child dyads, parent stress levels were similar to those that have been reported in previous studies using the parent distress subscale [20, 30, 31]. We found that parenting stress was not associated with measured child BMI, which is similar to the study that explored parenting stress and child weight status [13]. However, studies that have explored how general life stress (versus parenting-specific stress) influences child obesity risk have found a significant positive association. For example, Koch et al. [13] and Parks et al. [14] found that as a parent experienced more general stressors (e.g., financial), his/her child had an increased risk of obesity. These differing findings may be due to the use of different measures and definitions of stress among studies; because individuals have varying triggers and thresholds for stress, stress itself is difficult to define and measure using participant report [31]. These differing results may also suggest that general stressors have a stronger influence on child weight status than stress specific to parenting.

We found that the children of highly stressed parents were less likely to meet the physical activity recommendations in comparison to children with normally stressed parents. Participation in active play during the preschool years is associated with decreased adiposity, and improved measures of motor skill development, psychosocial health and cardio metabolic health [32, 33]. The majority of children in this study met the NASPE guidelines of participating in 60 minutes of moderate to vigorous activity per day (67% on weekdays, 73% on weekends) [23]. However, parenting stress was found to be associated with decreased participation in active play. This association existed only between parenting stress and weekday active play, suggesting that there are differences in the home environment between weekdays and the weekend. Parents may feel less stressed and have more free time during weekends to encourage and participate in physical activity. Future interventions designed to increase child physical activity could explore how addressing parenting stress, particularly on weekdays, may impact child activity levels.

While parenting stress levels were not associated with the amount of TV children were watching, parents who were experiencing high levels of stress, were less likely to set limits on the amount of time their children spent in front of the TV than parents who had normal levels of stress. Our findings are similar to those by Lampard et al. who found that less parenting stress and fewer life pressures were associated with greater screen time restriction among low-income families with preschoolers [16]. Previous studies have also found that maternal depression is associated with increased viewing times among young children [34, 35]. For parents to successfully monitor the amount of TV their children watch, it may be important to pay attention to the well-being of the parent and the role that the TV plays in managing parenting stress. While the children of parents who did not limit their child’s TV viewing did not spend any more time watching TV, a lack of limits may prove to be a problem as the child develops more independence. Adolescents who are not limited in their TV viewing as young children, may watch more TV than those who had specific limits placed on viewing time [36].

When interpreting the results of this study, limitations should be considered. First, as is common with secondary data analysis, the measurement of certain variables was not optimal. Only one subscale of the PSI-SF was used to measure parenting stress [37]. Using more than one approach to measure parenting stress would have provided a more thorough understanding of how different types of stress may influence obesity risk for preschoolers. Second, although, where possible, we used validated measures to assess our behavioral outcomes, we used parental report rather than objective measures, which may have introduced bias or random error. The message used to recruit families to the intervention study of learning tips on ‘raising happy, healthy preschoolers’ could have primed parents to answer questions in a way that made their children seem healthier. Third, based on the self-selection method of recruitment, it is possible that there may be systematic differences between those who choose to sign up for a parenting program and those that do not choose to sign up; parents who did sign up may have a heightened interest in or concern about their child’s health. This possible over-reporting of healthful behaviours caused both by the use of parental report measures and the self-selection bias of our recruitment methods may have biased our results towards our null hypothesis that parenting stress is not associated with child obesity risk or risk behaviours such as physical activity and TV viewing. Fourth, the results may not be generalizable to socioeconomically advantaged populations, as there are inherent demographic differences between our participants and the general U.S. population. While the inability to generalize the findings may be a limitation, ethnically diverse, low-income families were purposefully recruited as they bear a disproportionate burden of health related issues [29] and would benefit most from the PTT program. Fifth, the confidence intervals around our estimates for TV viewing were quite wide, suggesting that, due to our relatively small sample size, the null findings in this study could be the result of a Type II error. Finally, due to our cross-sectional study design, we are unable to determine changes over time. Parenting stress and parenting behaviours exhibited under stress may affect children’s obesity risk differently as they age.

Future research should be conducted with a larger sample of families not participating in a parenting program; stress levels may be different in those who are uninterested/unable to attend a 9-week program. Instead of self-report, biological, objective measures (saliva swabs or hair samples) should be used to quantify parental stress levels. Objective measures of child stress should also be taken to determine whether the child has experienced endocrine changes and metabolic disturbances that lead to weight gain. There is a need for longitudinal analysis to understand how parenting stress may affect obesity risk over time as well as the directionality of our findings. Furthermore, longitudinal analyses would allow us to understand how reduced parental TV limits affect the time preschoolers spend watching TV as they age.

## Conclusions

In summary, while previous research suggests that general stress is associated with childhood obesity risk, this study suggests that stress specific to parenting may not be an important focus in curbing the obesity epidemic. However, our results do suggest that interventions may need to address parental stress as an underlying factor associated with unhealthful behaviours among young children such as a lack of weekday active play and increased sedentary behaviours. Findings can help inform intervention messages and strategies to help parents of young children to promote healthful behaviours among their children.

## Abbreviations

BMI: Body Mass Index
TV: Television
PSI-SF: Parenting Stress Index Short Form
OR: Odds Ratio
CI: Confidence Interval
PTT: Parents and Tots Together
WHO: World Health Organization
NASPE: National Association of Sport and Physical Education
AAP: American Academy of Pediatrics
SD: Standard Deviation.
